# Calculation of Honeycomb Paperboard Resistance to Edge Crush Test

**DOI:** 10.3390/ma13071706

**Published:** 2020-04-06

**Authors:** Gabriela Kmita-Fudalej, Włodzimierz Szewczyk, Zbigniew Kołakowski

**Affiliations:** 1 Centre of Papermaking and Printing, Lodz University of Technology, Wólczańska 223, 90-924 Lodz, Poland; wlodzimierz.szewczyk@p.lodz.pl; 2 Department of Material Strength, Lodz University of Technology, Stefanowskiego 1/15, 90-924 Lodz, Poland

**Keywords:** honeycomb paperboard, edge crush test, critical load, mechanical properties

## Abstract

The article presents the method of calculating the edge crush test (*ECT)* of honeycomb paperboard. Calculations were made on the basis of mechanical properties of paper raw materials used for the production of cellular paperboard and geometrical parameters describing cellular paperboard. The presented method allows *ECT* calculation of honeycomb paperboard in the main directions in the paperboard plane; i.e., machine direction (*MD*) and cross direction (*CD*). The proposed method was verified by comparing the results of calculations with the results of *ECT* measurements of paperboard with different geometrical parameters made of different fibrous materials.

## 1. Introduction

Honeycomb board is manufactured on the basis of fibrous materials, most often from coniferous trees. Due to the spatial structure, it has low specific weight and good strength properties [1,2]. Its advantages also include recyclability; excellent energy absorption properties; and insulation, thermal, and acoustic properties [3]. Honeycomb paper cores are used in many multilayer and packaging products and they successively replace this type of plastic or aluminium products due to the lower manufacturing cost and lower specific weight. This organic and biodegradable raw material is gaining great popularity in various industries. It is used as a fillings for doors, countertops, furniture boards, partition walls in construction, and sandwiched multilayer structures in the aviation and automotive industries [4,5,6,7]. The production and use of honeycomb paper panels in the furniture industry are developing rapidly in Europe [8]. The demand for lighter furniture elements is increasing, which is contributing to lower transport costs and easier assembly, alongside the reduction of formaldehyde emissions, which is also an important issue in the modern world [9].

In the packaging industry, cellular paperboard is used to produce large-size boxes and their fillings. In most applications, in which cellular paperboard is treated as a construction material, it carries compressive loads caused by forces acting in its plane. To assess strength at this type of load, its edge crush test, *ECT,* can be used. In a certain direction along the paperboard plane, the *ECT* value is defined as the maximum compressive force transferred by the crushed paperboard until it is destroyed, related to the length of the side of the sample perpendicular to the direction of the force.

This indicator is particularly useful in cases wherein the strengths of panels with large values of panel thickness in the direction of load application are considered. This indicator is of decisive importance in, e.g., determining the resistance of boxes to the static pressure of boxes.

The study of cellular paperboard was carried out by Wen [10], who compared the results of measuring 5 mm thick cellular paperboard with the results of corrugated board. The comparison concerned both *ECT* edge crushing resistance in both directions in the paperboard plane, and the *FCT* flat crushing test.

Chen et al. [11] presented studies on lightweight multilayer panels with different honeycomb core structures made of paper, and wood cladding composite. By using experimental tests and finite element modelling methods, the authors presented the impacts of the construction parameters of honeycomb and the properties of the core material and cladding on the mechanical properties of light laminated panels.

Borsellino and Di Bella [12] conducted tests of laminates with different core structures at different load methods, including resistance to edge crushing of the paper honeycomb. The purpose of the work was based on experimental research to assess the relationship between stresses and deformations at uniform compressive static load.

Smardzewski et al. [13] conducted a study to determine the effect of a rectangular cellular paper core on the mechanical properties of three-layer furniture panels.

Smardzewski and Prekrat [14] presented modelling of mechanical properties of cellular wooden panels with a paper honeycomb core. The subjects of the study were the thin panels of a paper honeycomb with hexagonal cells. The research was carried out using numerical models; the results of numerical calculations were compared with the results of experimental measurements. As a result of the research, it was confirmed that the cores of cellular wood panels show strong orthotropic properties.

The authors of the work decided to develop a simple method for determining the *ECT* of honeycomb boards and to verify them in experimental studies. Another verification method was to develop complex numerical models in the finite element method (FEM), which should still be compared with experimental research.

Despite the fact that the literature provides information on *ECT* of honeycomb boards, none of the presented studies presents a simple, analytical method for determining the value of the edge crush resistance of cellular cardboard.

The purpose of the work is to present mathematical relationships that allow calculating the resistance of honeycomb paperboard to edge crushing in the machine and cross directions, based on its geometric parameters and mechanical properties of the materials from which it was made.

**Figure 1:**
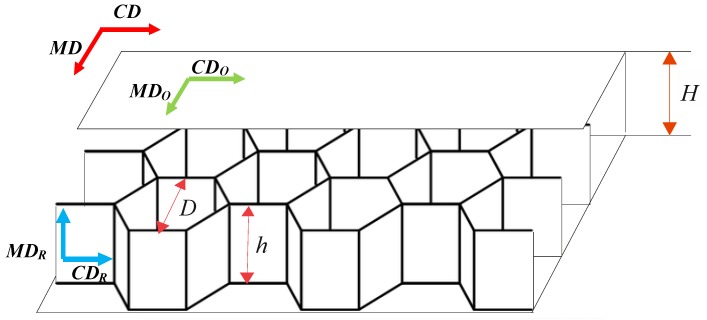
Cellular paperboard parameters.

Cellular paperboard consists of two outer layers and a honeycomb core (see Figure 1).

The geometrical parameters describing the cellular paperboard are:

*D*—diameter of the circle inscribed in the regular hexagon determined by the contact lines of the cell walls with cover layer, defined as the cell mesh size;

*h*—core height;

*H*—paperboard thickness.

**Figure 2:**
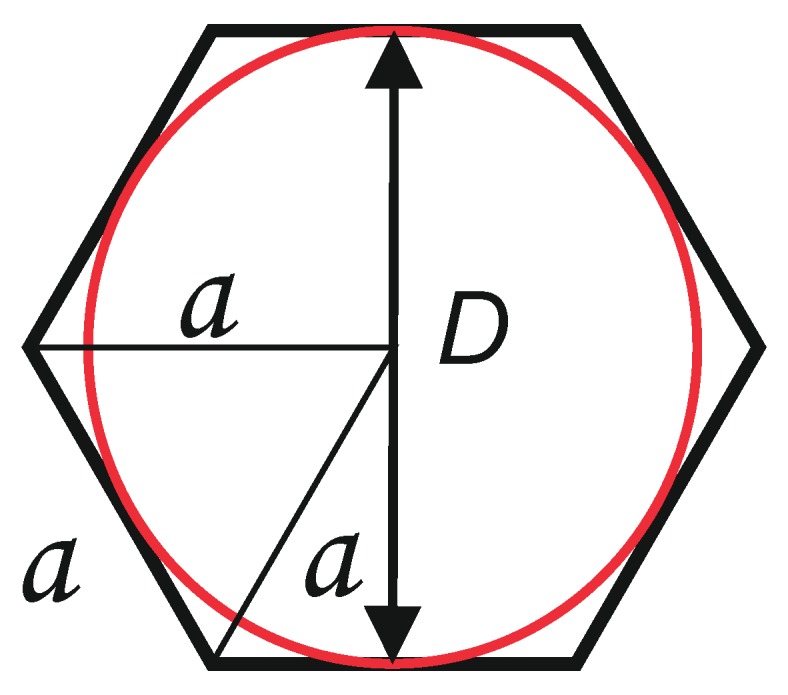
Cell mesh parameters.

The length of the regular hexagon’s side can be determined from Equation (1) using parameter *D* given by the paperboard manufacturer (see Figure 2):

(1) $$a=D/\sqrt{3}$$

Cellular cardboard has the characteristics of orthotropic bodies. This is due to the core structure and the distribution of mechanical properties of the flat layers, which is characteristic of orthotropic bodies. In the plane of cellular paperboard, two main directions of orthotropy can be distinguished. The first one coincides with the direction of manufacture and it is called machine direction, *MD*. The second main direction, perpendicular to the machine direction, is referred to as cross direction, *CD*. The main directions of the *CD* and *MD* of the paperboard coincide with the main directions of the paper used for the flat layers of the *CD_O_* and *MD_O_* paperboard (see Figure 1). In the case of a paperboard core, the machine direction of the paper used for the *MD_R_* core is parallel to the height of the core and the cross direction *CD_R_* is perpendicular to the height of the core.

The proposed method for determining the resistance of honeycomb paperboard to edge crushing is based on the stability of thin-walled isotropic [15,16,17,18] and orthotropic plates [19,20,21,22,23]. The formulas for calculating *ECT* presented in the article were developed in detail in [24,25,26].

## 2. Materials and Methods

Sixteen honeycomb paperboards with different geometrical parameters and made of different fibrous materials were tested, and 4 types of paper used for their production.

The following papers are used in the remainder of the article:

- T135—testliner, 135 g/m^2^ basis weight;
- T160—testliner, 160 g/m^2^ basis weight;
- T200—testliner, 200 g/m^2^ basis weight;
- F140—fluting, 140 g/m^2^ basis weight.

Table 1 presented material constants of the papers.

The honeycomb paperboard markings contain information about their material composition according to the following record (material of the first cover layer/core material/material of the second cover layer). For example: honeycomb paperboard with the core made of T160 paper and two cover layers of T135 paper has the mark T135/T160/T135.

To identify each paperboard, thickness *H*, the diameter of the circle inscribed in the regular hexagon of the core *D* cells, and the markings of the paper from which it was made, are provided.

To avoid the impact of climatic conditions on the results of strength tests of papers and boards, the testing pieces were conditioned before the test in accordance with PN-EN 20187: 2000 [27], and the tests were carried out in an air-conditioned room with the same climatic conditions as during the conditioning of the testing pieces; i.e., temperature 23 ± 1 °C and relative air humidity 50% ± 2%.

In the case of paper from which paperboard was made, the basis weight was measured in accordance with PN-EN ISO 536: 2012 [28], thickness in accordance with PN-EN ISO 534: 2012 [29], and the Young’s modulus in machine and cross directions was determined based on a tensile test at constant speed stretching performed in accordance with PN-EN ISO 1924-2: 2010 [30].

**Figure 3:**
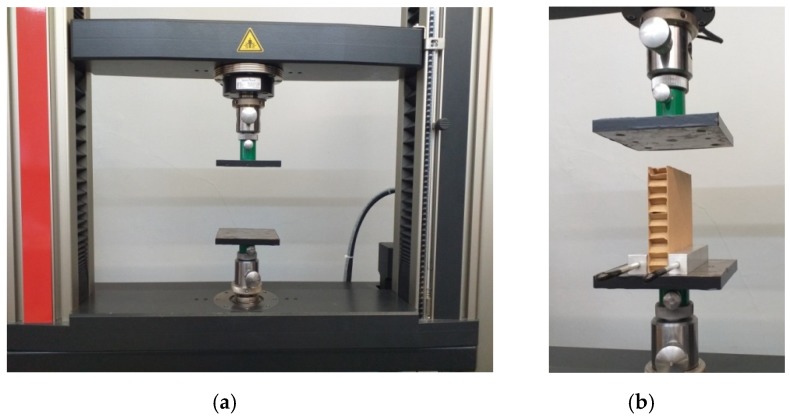
Instrumentation for the edge crush test (*ECT)*: (**a**) measuring instrumentation; (**b**) test sample supported by support blocks.

*ECT* measurements were made using a universal Zwick testing machine (Ulm, Germany) with a load range up to 20 kN using the tooling shown in Figure 3. The tooling consists of two square plates of 144 cm^2^ each. Both plates are rigidly attached to the lower frame of the machine, and the upper to the movable traverse (see Figure 3a). Supporting blocks (see Figure 3b) were used to test cardboard with small thickness, maintaining the tested piece in a vertical position until reaching the initial force.

One-hundred-millimetre squared tested pieces were crushed; only for paperboard less than 10 mm thick were the tested pieces reduced to 50 mm to protect against global buckling.

Before starting the measurement, the tested piece was subjected to a 10 N initial force. During the measurements, the plates approached each other at a speed of 12.5 mm/min.

The measurements were carried out in two main directions in the plane of the paperboard; and on their basis its resistance to edge crushing in the machine direction *ECT_MD_* and cross direction *ECT_CD_* was determined respectively. The result of the determination in each direction is given as the average value obtained after testing ten pieces.

The *ECT* value was calculated from the equation:

(2) $$ECT=\frac{F}{l},\;kN/m$$

where:

*F*—value of destructive force, kN;
*l*—length of the loaded edge of the tested piece, m.

### Calculation Methodology

The resistance of cellular board, both in the machine direction and in the cross direction, was calculated as the sum of loads carried by the core and both cover layers:

(3) $$ECT_{MD}=ECT_{RMD}+ECT_{OMD}$$

(4) $$ECT_{CD\;}=ECT_{RCD}+ECT_{OCD}$$

where:

*ECT_RCD_* and *ECT_OCD_*—edge crushing resistance towards *CD*, core and both cover layers, respectively,
*ECT_RMD_* and *ECT_OMD_*—resistance to edge crushing in the *MD* direction of core and both cover layers, respectively.

**Figure 4:**
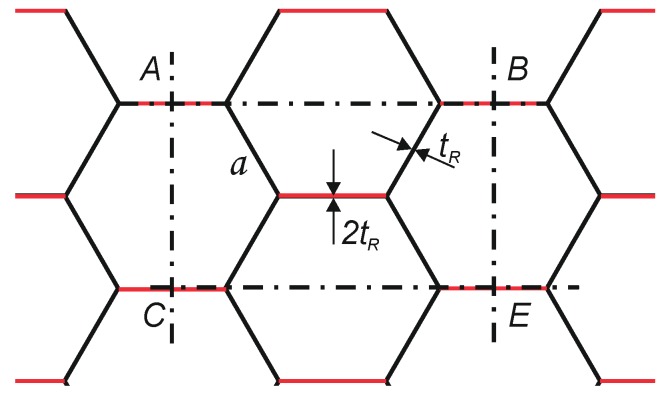
*ABCE* periodic cell extracted from the paperboard core.

It was assumed that the calculation model of the resistance to edge crushing of the core will describe the destruction of a repeating element of the paperboard core structure. The *ABCE* periodic cell was separated from the honeycomb core structure (see Figure 4).

It has been assumed that the cross-section of the core cell with a plane parallel to the cover layer has the shape of a regular hexagon with side *a*.

**Figure 5:**
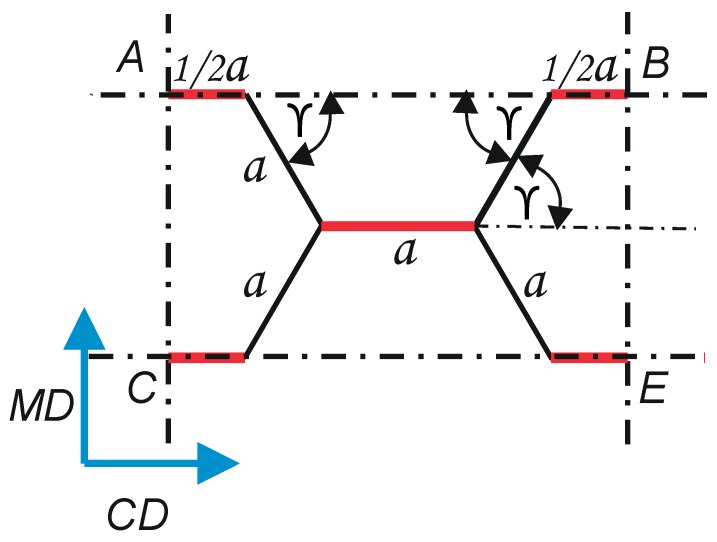
Dimensions of the periodic core cell.

Figure 5 shows the dimensions of the periodic cell.

The dimensions of the periodic cell can be determined from the equation:

(5) CE=AB=2·a+2·a ·cosγ

(6) AC=BE=2·a ·sinγ

In the case of a regular hexagon cell that has been taken into account in the calculations, the angle *γ* is 60°. It was assumed that only single-thickness walls are responsible for cell destruction in the *ECT* test, *t_R_* marked in black in Figure 4. The double walls *t_R_*, which are formed by gluing two layers of core material marked in red in Figure 4, are not damaged, as observed in preliminary *ECT* tests of honeycomb panels.

**Figure 6:**
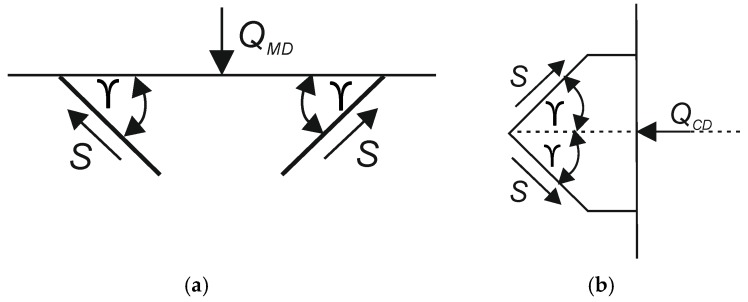
Load diagrams used to calculate *Q* forces: (**a**) in machine direction (*MD)*, (**b**) towards cross direction (*CD)*.

The load schemes used to calculate the *Q* forces transmitted through the periodic core cell in the *MD* and *CD* directions are illustrated in Figure 6.

The maximum *Q_MD_* and *Q_CD_* forces transferred by the periodic core cell in the *MD* and *CD* direction were calculated from:

(7) $$Q_{MD\;}=2\;\cdot S\;\cdot \sin\gamma$$

(8) $$Q_{CD\;}=2\;\cdot S\;\cdot \cos\gamma$$

where:

*S*—maximum force transmitted during compression in the cross direction of the core material by a wall of a single thickness.

The force *S* can be determined from the following relationship:

(9) $$S=\alpha \;\cdot \;t_{R}\;\cdot h\;\cdot \;\sigma_{cr}$$

where:

*t_R_*—thickness of paper used for core production;
*h*—core thickness of cellular paperboard;
*σ*_cr_—critical stress;
*α*—coefficient of elastic restoration of a single cell wall.

The α factor is respectively:

(10) $$\alpha =1.0\;\;gdy\;\;\frac{a}{h}>1.0$$

(11) $$\alpha =3.41-1.41\frac{a}{h}\;\;gdy\;\;0.2\leq \frac{a}{h}\;\leq 1.0$$

(12) $$\alpha =1.0\;\;gdy\;\;0.1<\frac{a}{h}<0.2\;$$

Critical stress *σ*_cr_ can be determined from the equation:

(13) $$\sigma_{cr}=\frac{\pi^{2\;}\cdot \;{t_{R}}^{2}}{12\;\cdot \;h^{2}}\sqrt{E_{RMD}\cdot E_{RCD}}\left[\eta +2+\frac{1}{\eta }\right]$$

where:

(14) $$\eta ={\left(\frac{h}{a}\right)}^{2}\cdot \sqrt{\frac{E_{\mathrm{RCD}}}{E_{\mathrm{RMD}}}}$$

Substituting Equation (14) into Equation (13) gets:

(15) $$\sigma_{cr}=\frac{\pi^{2}\;\cdot \;t_{R}^{2}}{12\;\cdot \;h^{2}}\;\cdot \;\sqrt{E_{RMD}\;\cdot \;E_{RCD}\;}\cdot \;\left[2+{\left(\frac{h}{a}\right)}^{2}\;\cdot \;\sqrt{\frac{E_{RCD}}{E_{RMD}}}+\frac{1}{{\left(\frac{h}{a}\right)}^{2}\;\cdot \;\sqrt{\frac{E_{RCD}}{E_{RMD}}}}\right]$$

where:

*E_RCD_*, *E_RMD_*—Young’s paper moduli used to produce the cellular paperboard core in cross and machine directions, respectively.

Many studies are devoted to a detailed discussions of Equations (13) and (14), as presented by Kołakowski et al. [24,25,26].

The *ECT_RMD_* and *ECT_RCD_* core crush test was calculated from the relationship:

(16) $$ECT_{RMD}=\frac{Q_{MD}}{AB}=\frac{\alpha \;\cdot \;t_{R\;}\;\cdot \;h\;\cdot \;\sigma_{cr}\;\cdot \sin\gamma }{a\;\cdot \;\left(1+cos\;\gamma \right)}$$

(17) $$ECT_{RCD}=\frac{Q_{CD}}{AC}=\frac{\alpha \;\cdot \;t_{R\;}\;\cdot \;h\;\cdot \;\sigma_{cr}\;\cdot \cos\gamma }{a\;\cdot \;sin\;\gamma }$$

The resistance of two honeycomb board cover layers to *ECT_OMD_* and *ECT_OCD_* edge crushing was determined from the following equation:

(18) $$ECT_{OMD}=\beta_{OMD}\;\cdot \;\frac{2\;\cdot \;\pi^{2}\;\cdot \;t_{o}^{3}}{3\;\cdot \;{\left(a+2\;\cdot a\;\cdot cos\gamma \right)}^{2}}\;\cdot \;\sqrt{E_{OMD}\cdot E_{OCD}}$$

(19) $$ECT_{OCD}=\beta_{OCD}\;\cdot \;\frac{\pi^{2}\;\cdot \;t_{o}^{3}}{6\;\cdot \;{\left(\;a\;\cdot sin\gamma \right)}^{2}}\;\cdot \;\sqrt{E_{OMD}\cdot \;E_{OCD}}$$

where:

*E_OCD_, E_OMD_*—Young’s moduli of paper used to produce cover layers of cellular paperboard in cross and machine directions, respectively;
*t_o_*—the thickness of the paper used for flat layers of cellular paperboard;
*β_OMD_*, *β_OCD_*—elastic support coefficients, determined experimentally.

It should be taken into account that *E_OMD_ ≠ E_OCD_*, and this implies that *β_OMD_* *≠ β_OCD_*.

The *β_OMD_* factor is assumed as a function of the *a/h* ratio:

(20) $$\frac{a}{h}\leq 0.6\;\cdot \sqrt[{4}]{\frac{E_{OMD}}{E_{OCD}}}\;\;to\;\;\beta_{OMD}=1.1\;\cdot \;\sqrt[{4}]{\frac{E_{OMD}}{E_{OCD}}}$$

(21) $$\frac{a}{h}>0.6\;\cdot \sqrt[{4}]{\frac{E_{OMD}}{E_{OCD}}}\;\;to\;\;\beta_{OMD}=\left[1.1+0.3\;\cdot \;\left(\frac{a}{h}\right)\right]\cdot \;\sqrt[{4}]{\frac{E_{OMD}}{E_{OCD}}}$$

The coefficient *β_OCD_* is constant, and it is:

(22) $$\beta_{OCD}=\left[1.5+0.6\left(\frac{a}{h}\right)\right]\cdot \sqrt[{4}]{\frac{E_{OCD}}{E_{OMD}}}$$

The cellular board’s resistance to edge crushing in the machine direction *ECT_MD_* and cross direction *ECT_CD_* can be determined from the Equations (23) and (24), obtained after substituting Equations (16) and (18) into Equations (3) and (17), and Equation (19) to Equation (4):

(23) $$ECT_{MD}=ECT_{RMD}+ECT_{OMD}=\alpha \cdot \;\frac{t_{R}\cdot \;h\;\cdot \sigma_{cr\;}\cdot sin\gamma \;}{a\;\cdot \left(1+cos\gamma \right)}+\beta_{OMD}\cdot \frac{2\;\cdot \;\pi^{2}\;\cdot \;t_{o}^{3}}{3\;\cdot \;{\left(a+2\;\cdot a\;\cdot cos\gamma \right)}^{2}}\;\cdot \sqrt{E_{OMD}\cdot \;E_{OCD}}$$

(24) $$ECT_{CD}=ECT_{RCD}+ECT_{OCD}=\alpha \cdot \;\frac{t_{R}\cdot \;h\;\cdot \sigma_{cr\;}\cdot cos\gamma \;}{a\;\cdot sin\gamma }+\beta_{OCD}\cdot \frac{\pi^{2}\;\cdot \;t_{o}^{3}}{6\;\cdot \;{\left(a\;\cdot sin\gamma \right)}^{2}}\;\cdot \sqrt{E_{OMD}\cdot \;E_{OCD}}$$

## 3. Results and Discussion

Theoretical *ECT* values in the machine direction *ECT_MD_* and cross direction *ECT_CD_* were calculated based on the results of measurements of the properties of the materials used for the production of cellular paperboards and the geometrical parameters of the boards.

Table 2 and Table 3 summarise the results of *ECT* measurements and calculations in both main directions.

**Figure 7:**
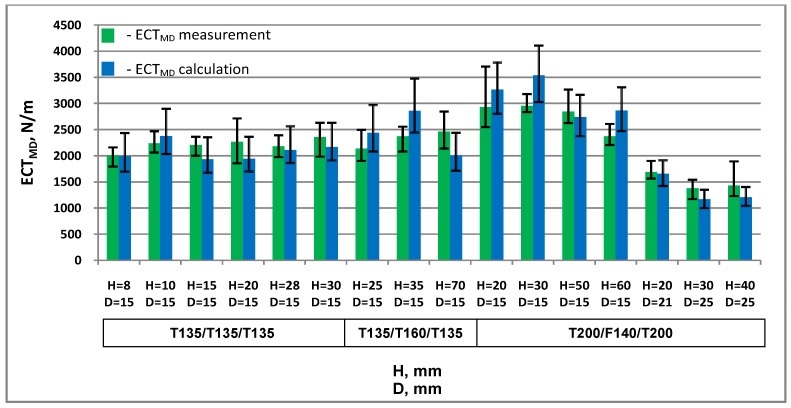
Resistance of honeycomb paperboard to edge crushing in the machine direction.

**Figure 8:**
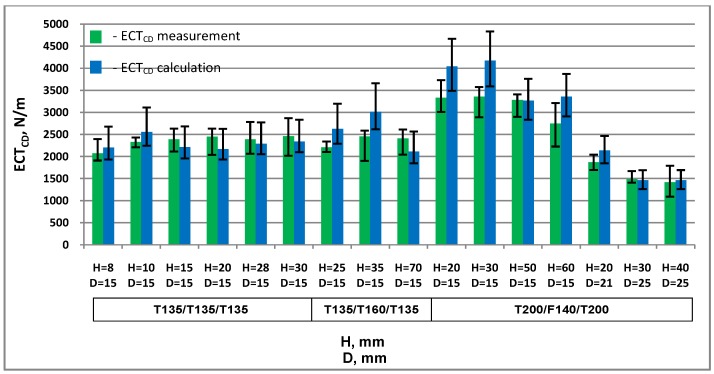
Resistance of honeycomb paperboard to edge crushing in the cross direction.

Figure 7 and Figure 8 show a comparison of measurement results and calculations of cellular board resistance to edge crushing in the machine and cross directions.

In almost all cases examined, the differences between the measured and calculated *ECT* values are within the variability of the results of measurements and calculations. For two honeycomb paperboards, the minimum calculation value is greater than the maximum value obtained from measurements by less than 1.2%.

The largest discrepancy between the measured and calculated values was in the *MD* direction: 20% of the actual *ECT_MD_* value and in the *CD* direction 24%. The largest *ECT_MD_* discrepancy was found in the case of cardboard with mesh size *D* = 15 mm, thickness *H* = 60 mm, made of T200 and F140 papers. The largest discrepancy between the calculated and measured *ECT_CD_* values was in the case of paperboard with mesh size *D* = 15 mm, thickness *H* equal to 30 mm, made of T200 and F140 papers. The mean value of the discrepancy between the measured and calculated *ECT* values in all the cases examined was 11% of the actual value in both *MD* and *CD*.

**Figure 9:**
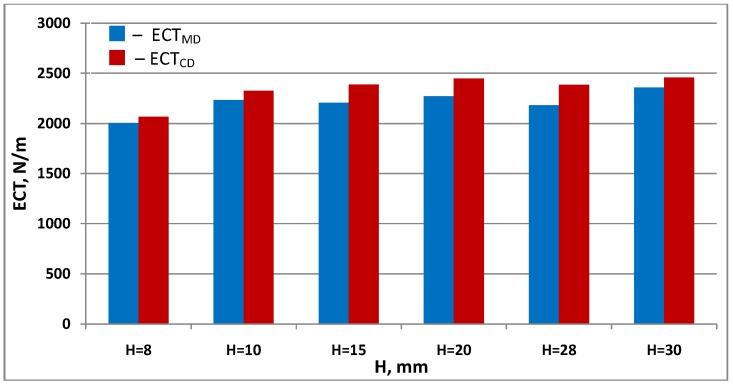
Results of *ECT_MD_* and *ECT_CD_* measurements of paperboard with 15 mm mesh diameter, made of T135 paper.

Figure 9 summarizes the *ECT* measurements of paperboard with the same mesh size made of the same materials.

In both cases, at low core heights, up to 20 mm, there is an increasing tendency for greater edge crush resistance as the cardboard thickness increases. Then, the *ECT* value remains at the same level. This is due to the fact that as the value of the *a/h* decreases, the influence of the core on the *ECT* of the paperboard decreases, striving for a constant value.

**Figure 10:**
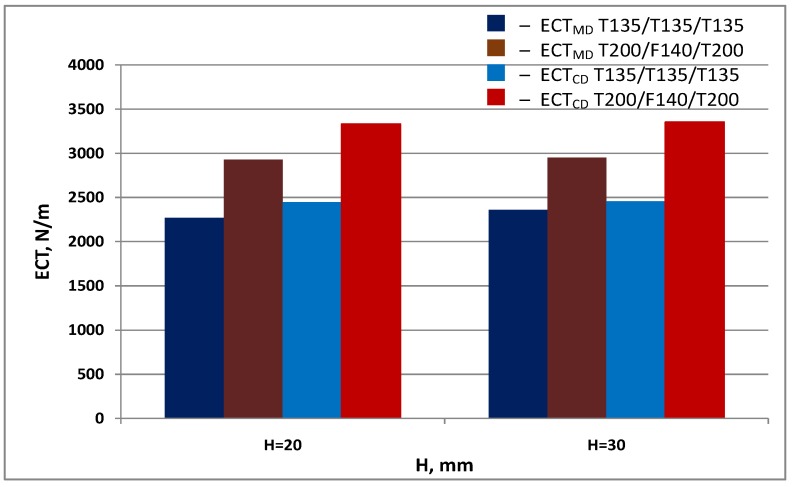
Results of *ECT_MD_* measurements of cardboard with a 15 mm mesh diameter, made of various raw materials.

Figure 10 shows *ECT* values of cardboard with the same geometrical parameters made of various raw fibres.

The measurement results illustrated in Figure 10 show the effect of physical properties of the papers on the *ECT* of cellular board. The use of a paper with a higher basis weight, and thus higher thickness and better mechanical properties, causes a significant increase in the *ECT* value in both machine and cross directions.

**Figure 11:**
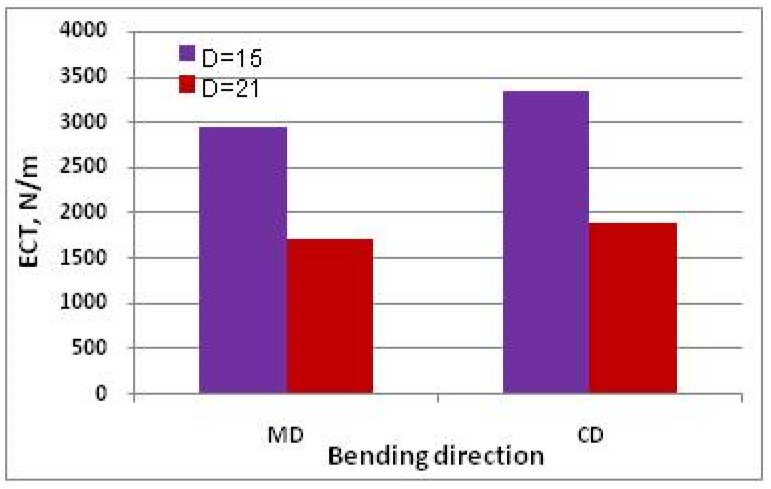
*ECT* of paperboard with thickness *H* = 20 and different mesh sizes, made of paper T200/F140/T200.

**Figure 12:**
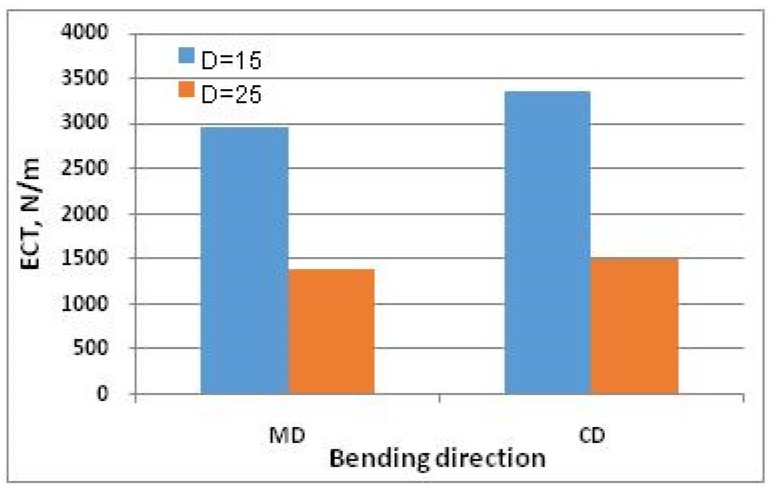
*ECT* of paperboard with thickness *H* = 30 and different mesh sizes, made of T200/F140/T200 papers.

Figure 11 and Figure 12 show *ECT* values of paperboard made from the same raw materials of the same thickness, differing in mesh size.

The very large decrease in *ECT* of cellular board visible in Figure 11 and Figure 12 is associated with a decrease in the force transmitted by the cover layers, which buckle more easily with an increase in mesh size.

The relationships between the individual parameters used to calculate *ECT* and its value show a consistent nature with the mathematical relationships presented in Equations (23) and (24); e.g., an increase in cardboard thickness causes an increase in *ECT* value, while an increase in mesh size causes a decrease in *ECT* value.

The big impact on the differences between the real values of edge crush resistance and the values calculated in theoretical way result from the fact that the paperboard was produced in different periods of time, and during the production of the core, the papers are unwound simultaneously from several turns, and thus for their production, materials from various supplies are used, the mechanical properties of which may differ significantly. It happens that the actual values of mechanical properties differ by up to 20% from the nominal values given in the specification. In addition, switching machines for the production of paperboard of a different thickness can cause a different degree of stretching of the core or a different arrangement of the cell wall gluing lines, and thus the deviation of the dimensions and shape of the cell from the cells shaped like a hexagon, which is the shape adopted in the mathematical description. Very often during production, the core is slightly crushed, which also has a significant impact on the value of edge crush resistance [31].

During the tests, no global buckling of paperboard samples was found. Shibao Wen [10], who tested much thinner (and thus more vulnerable to global buckling) cellular cardboard with a thickness of about 5 mm did not find this phenomenon.

## 4. Conclusions

The proposed method allows one to calculate the *ECT* of cellular paperboard both in machine and cross directions on the basis of the paperboard’s geometric parameters and the mechanical properties of materials used for its production. It is much easier and much faster in practical application than numerical methods such as the finite element method or the finite difference method. In the examined range of *a/h* values in each of the main directions in the board plane, the theoretically calculated values differed from real values by an average of 11% of the actual value, and the maximum difference that occurred in the cross direction was 24%. Considering that the mechanical properties of raw materials and the geometrical parameters of paperboard can differ significantly from the nominal values assumed during the calculations, the obtained calculation accuracy can be considered satisfactory. In the future, the authors plan to compare the results obtained with the proposed calculation method and the results obtained by numerical calculations. However, they do not expect significant differences in the accuracy of the calculations due to the fact that the calculation errors result from the variability of strength properties of materials and heterogeneity of the geometry of the core due to changes in the parameters of the production process.

## Figures and Tables

**Table 1:** Material constants of the papers are presented.

Symbol	Paper Thickness (mm)	Young’s Modules of Paper in Cross Direction (GPa)	Young’s Modules of Paper in Machine Direction (GPa)
T135	0.204	1.8	5.3
T160	0.209	2.3	4.9
T200	0.263	2.3	5.7
F140	0.203	2.1	5.5

**Table 2:** *ECT* measurement results: means of ten measurements and standard deviations.

Symbol	*D* mm	*H* mm	*ECT_MD_* N/m	Max *ECT_MD_* N/m	Min *ECT_MD_* N/m	SD_MD_ N/m	*ECT_CD_* N/m	Max *ECT_CD_* N/m	Min *ECT_CD_* N/m	SD_CD_ N/m
T135/T135/T135	15	8	2005	2159	1794	159	2067	2392	1906	187
T135/T135/T135	15	10	2235	2468	2061	115	2326	2429	2208	97
T135/T135/T135	15	15	2207	2362	2000	84	2389	2630	2110	215
T135/T135/T135	15	20	2270	2713	1854	306	2447	2629	2034	188
T135/T135/T135	15	28	2182	2390	1972	187	2387	2781	2058	258
T135/T135/T135	15	30	2358	2630	1982	336	2457	2866	2016	340
T135/T160/T135	15	25	2138	2496	1900	182	2204	2337	2100	103
T135/T160/T135	15	35	2369	2555	2081	188	2454	2586	1897	182
T135/T160/T135	15	70	2464	2844	2136	217	2409	2608	2039	196
T200/F140/T200	15	20	2930	3706	2550	321	3334	3724	3005	391
T200/F140/T200	15	30	2953	3177	2834	110	3355	3569	2887	238
T200/F140/T200	15	50	2843	3264	2623	247	3281	3403	2894	103
T200/F140/T200	15	60	2370	2608	2203	170	2745	3210	2223	314
T200/F140/T200	21	20	1691	1901	1562	157	1872	2042	1691	159
T200/F140/T200	25	30	1378	1538	1169	118	1487	1665	1406	122
T200/F140/T200	25	40	1433	1891	1227	191	1417	1790	1089	276

**Table 3:** *ECT* calculation results.

Symbol	*D* mm	*H* mm	*ECT_MD_* N/m	Max *ECT_MD_* N/m	Min *ECT_MD_* N/m	*ECT_CD_* N/m	Max *ECT_C_*_D_ N/m	Min *ECT_CD_* N/m
T135/T135/T135	15	8	1989	2434	1692	2200	2675	1928
T135/T135/T135	15	10	2373	2897	2033	2554	3105	2240
T135/T135/T135	15	15	1932	2353	1675	2207	2679	1956
T135/T135/T135	15	20	1942	2363	1697	2163	2624	1929
T135/T135/T135	15	28	2111	2564	1859	2286	2771	2051
T135/T135/T135	15	30	2166	2631	1911	2334	2828	2096
T135/T160/T135	15	25	2436	2968	2080	2625	3191	2285
T135/T160/T135	15	35	2856	3476	2442	3010	3656	2615
T135/T160/T135	15	70	2004	2439	1712	2111	2563	1844
T200/F140/T200	15	20	3265	3780	2803	4040	4665	3485
T200/F140/T200	15	30	3539	4106	3028	4171	4828	3584
T200/F140/T200	15	50	2742	3165	2374	3261	3756	2830
T200/F140/T200	15	60	2864	3309	2474	3355	3869	2905
T200/F140/T200	21	20	1654	1915	1421	2136	2464	1845
T200/F140/T200	25	30	1166	1350	1002	1460	1686	1261
T200/F140/T200	25	40	1209	1402	1037	1461	1690	1259
